# Improved and simplified method for aseptic isolation of nematodes and nematode-endosymbiotic bacteria from pine seedlings

**DOI:** 10.1016/j.mex.2023.102421

**Published:** 2023-10-07

**Authors:** Mohamed Mannaa, Young-Su Seo

**Affiliations:** a Department of Integrated Biological Science, Pusan National University, Busan 46241, Republic of Korea; b Department of Plant Pathology, Cairo University, Faculty of Agriculture, Giza 12613, Egypt

**Keywords:** Pine wood nematode, Bursaphelenchus xylophilus, Nematode-associated bacteria, Aseptic Isolation and Extraction of *B. xylophilus* and endosymbiotic bacteria from Pine Seedlings

## Abstract

Pine wilt disease (PWD), caused by the pinewood nematode (PWN), *Bursaphelenchus xylophilus*, significantly impacts pine species and poses a broader ecological concern. An understanding of these nematode-associated microbes is essential for formulating sustainable PWD management strategies. We introduce a streamlined method for the aseptic extraction of *B. xylophilus* from pine seedlings, evolving beyond traditional Baermann funnel approaches. The method ensures optimal nematode extraction under sterile parameters, with seedling cutting discs processed using a unique sterile syringe assembly setup. The efficiency and simplicity of this method promise to significantly reduce the time and resources required. It also incorporates endosymbiotic bacterial isolation from isolated nematodes. The robustness of this method is affirmed by the successful isolation and identification of nematodes and bacterial strains as endosymbionts. Collectively, this protocol paves the way for more effective studies of nematodes and associated microbes, promoting the understanding of PWD and offering practical implications for better PWD management.

• A simplified, aseptic method for extracting *B. xylophilus* from pine seedlings, offering a modern alternative to traditional Baermann funnel method.
• Utilization of a specialized sterile syringe assembly setup, ensuring controlled and optimal nematode isolation.
• Method validation achieved through the successful isolation and identification of bacterial strains as nematode endosymbionts.

## Graphical abstract

**Figure:**
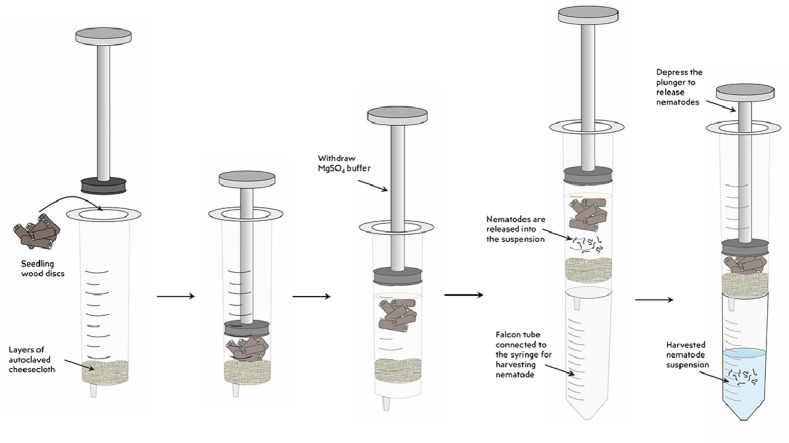

Specifications Table

**Table:** 

Subject area:	Agricultural and Biological Sciences
More specific subject area:	Nematology and Plant Pathobiome
Name of your method:	Aseptic Isolation and Extraction of *B. xylophilus* and endosymbiotic bacteria from Pine Seedlings
Name and reference of original method:	Baermann Funnel Technique - Baermann, G. (1917). Eine einfache Methode zur Auffindung von Ankylostomum (Nematoden) Larven in Erdproben. Geneesk. Tijdschr. Ned-Indië 57: 131-137.
Resource availability:	N.A.

## Method details

### Background

Pine wilt disease (PWD), primarily attributed to the pine wood nematode (PWN), *Bursaphelenchus xylophilus*, has critically impacted pine forests, especially in East Asia [1]. While PWN is the primary agent, its associated microbiota is increasingly believed to play a pivotal role in the disease progression, necessitating a thorough understanding for effective PWD management [2,3]. The complex relationships between PWN and its associated microbes underscore the necessity for precise and contamination-free isolation methods. As molecular techniques and genomic analyses unveil the multifaceted interactions between the organisms involved in PWD, it becomes important to understand the roles of nematode-carried bacteria. Their exact role, whether in synergistic damage with the PWN or in potentially controlling the nematode population within the tree, is still a subject of ongoing research [3,4]. Therefore, methods ensuring the accurate study of both nematodes and their associated microbiota can provide important insights into the etiology and management of PWD.

Historically, nematode isolation has relied on the Baermann funnel method, a technique in use for over a century [5]. This classic technique involves a funnel outfitted with a rubber tube and clamp, complemented by a sieve lined with a filtering material, like cheesecloth. To extract nematodes, the sample material is placed on the sieve, and water is introduced, partially submerging the material. As nematodes navigate out of the sample, they pass through the sieve and accumulate at the clamped end of the tubing, from which they can be collected by unclamping and draining the water into a collection vessel [6]. While effective to an extent, the traditional Baermann funnel method poses several limitations: potential contamination, lower precision for specific contexts such as isolating nematodes from tree tissues, and the inability to maintain aseptic conditions. In recognition of the limitations of the method, various adaptations, such as the mini-Baermann funnel, have been introduced to enhance its efficiency [7]. Despite the widespread adoption of Baermann funnel and derivatives, there is a growing need for a method that offers more precision, especially for isolating nematodes from trees and seedling woods tissues in aseptic conditions. Therefore, this study aimed to introduce a method that overcomes the limitations of traditional nematode isolation techniques, allowing for precise and aseptic extraction of PWN from pine seedlings. This modern approach can lead to refined studies on nematode-associated microbiota, which in turn can facilitate our understanding of PWD and offer more sustainable countermeasures [1].

As PWD continues to pose significant environmental and economic challenges, exacerbated by the looming threats of climate change [8], the method explained herein presents a novel approach for simplified, controlled and aseptic isolation of PWN from pine seedlings, aiming to enrich our understanding for management of this detrimental disease.

## Materials

• Pine wood nematode (PWN) *B. xylophilus*, isolated from pine wilt disease infected trees, were obtained from the National Institute of Forest Research in Seoul, South Korea.
• Pine seedlings (*Pinus densiflora*) 3 years old.
• Culture of *Botrytis cinerea.*
• 70 % ethanol.
• Sterile distilled water (SDW).
• Sterile plant stem cutting scissors.
• 50 mL sterile surgical syringes.
• Autoclaved cheesecloth (4 layers).
• Sterile 50 mL Falcon tubes.
• Plastic wrap.
• 1 % sodium hypochlorite.
• Nutrient agar plates (Difco; Becton, Dickinson and Company, Sparks, MD, United States).
• Potato dextrose agar plates (Difco; Becton, Dickinson and Company, Sparks, MD, United States).
• Sterilized 10 mM MgSO_4_ buffer solution.

### Preparation and Sterilization of Materials

• 4-layer pieces (∼4 cm^2^) of cheesecloth were cut and wrapped in aluminum foil before being placed in an autoclave. They were then sterilized using a standard autoclave cycle at 121 °C for 15 min.
• After sterilization, the cheesecloth pieces were transferred into the syringe barrel using sterilized forceps.

### Method and Procedure

#### The *in planta* seedling assay for the development of pine wilt disease

The PWN was grown on the mycelia of the fungus *Botrytis cinerea* on PDA. The cultures were maintained at 25 °C in the dark for a duration of 1 week. PWN was then extracted from the medium and used for inoculation in the seedling assays. For the *in planta* seedling assays, *P. densiflora* seedlings, between 40–50 cm in height and 0.5–1.0 cm in diameter, were acquired from the Daelim seedling farm (Okcheon, South Korea). The seedlings were then relocated to 15 cm diameter pots filled with sterilized nursery soil. The relocated seedlings were housed in a greenhouse where conditions were controlled to an average temperature of 25 °C and 70% relative humidity. Pine seedlings underwent inoculation with PWN, following the method presented in our earlier study [9]. A PWN aqueous suspension, consisting of 2,000 nematodes/100 µL, was carefully pipetted onto sterile cotton that was positioned within a minor incision made on the pine seedlings using a surface-sterilized blade. To maintain moisture and prevent dehydration, the cotton with the nematode suspension was subsequently sealed with Parafilm M (Heathrow Scientific, Vernon Hills, IL, USA). Inoculated seedlings were used for the nematode isolation from infected tissues. Five seedlings were used for inoculation and the control, and the experiments was conducted twice for validation of the method.

#### The modified method for the isolation of nematodes from the inoculated seedlings

1. Using the sterile plant cutting scissors, carefully cut the pine seedlings, avoiding the inoculation curt where the seedlings' stems were previously inoculated with the nematode *B. xylophilus*.
2. Harvest a roughly 5 to 10 cm sections of the seedling stem 2 cm above and below the infection curt. Surface sterilize this section by immersing it in 70  % ethanol for 2 min, followed by three rinses in SDW.
3. Subsequently, cut the surface-sterilized stem sections into smaller discs, each approximately 0.5 cm in length, using sterile plant stem cutting scissors.
4. Prepare a 50 mL sterile surgical syringe by first removing the plunger. Then, place a 4-layer piece of autoclaved cheesecloth inside the syringe barrel.
5. Aseptically transfer the stem pieces on top of the cheesecloth within the syringe barrel.
6. Re-insert the plunger at the top and gently push to compact the stem pieces.
7. Aseptically withdraw 25 mL of sterilized 10 mM MgSO_4_ solution into the syringe containing the stem pieces.
8. Attach the syringe to a sterile 50 mL Falcon tube. Wrap the assembly with plastic wrap to maintain sterility.
9. Place the setup in a vertical position and let it sit for 8 h in the dark.
10. After 8 h, gently depress the syringe plunger to release the solution, which now contains the nematodes, into the Falcon tube.
11. To ensure complete nematode recovery, draw up an additional 25 mL of sterilized 10 mM MgSO_4_ into the syringe, let it sit for 2 h and gently press the plunger to release the solution into the Falcon tube.

**Fig. 1:**
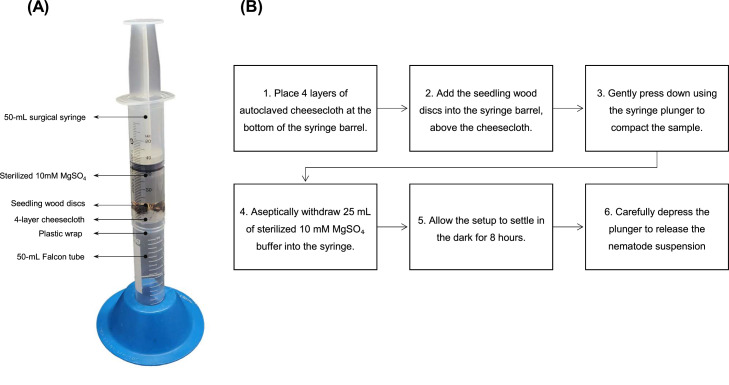
(A) The syringe-based setup designed for the aseptic isolation of nematodes from pine seedling tissues. (B) Flowchart illustrating the step-by-step process of nematode isolation using our improved method.

A detailed illustration of the syringe-based setup and a flowchart highlighting the associated steps involved in the process of nematode isolation and the use of the syringe setup are shown in Fig. 1.

### Surface sterilization and isolation of nematode endo-symbiotic bacteria

1. Centrifuge the solution containing the nematodes at 3000 *g* for 5 min to pellet the nematodes, then carefully discard the supernatant.
2. Add 1  % sodium hypochlorite to the nematode pellet for surface sterilization, mix gently for 1 min.
3. Wash the nematodes by centrifugation, three times with 10 mM MgSO_4_ solution, discarding the supernatant after each wash.
4. Resuspend the washed and surface-sterilized nematode pellets in an appropriate volume of 10 mM MgSO_4_ solution and perform a count.
5. Surface sterilization is verified by spreading 100 µL of the nematode suspension onto a NA plate without grinding the nematodes.
6. Grind the verified surface-sterilized nematodes into a homogenate in sterile mortars under sterile conditions.
7. Spread 100 µL of the nematode homogenate onto NA plates.
8. Incubate the plates at 28 °C until colonies appear.
9. Select colonies with different morphologies and growth rates for further purification into pure cultures.
10. Store the purified cultures under appropriate conditions for future use.

**Fig. 2:**
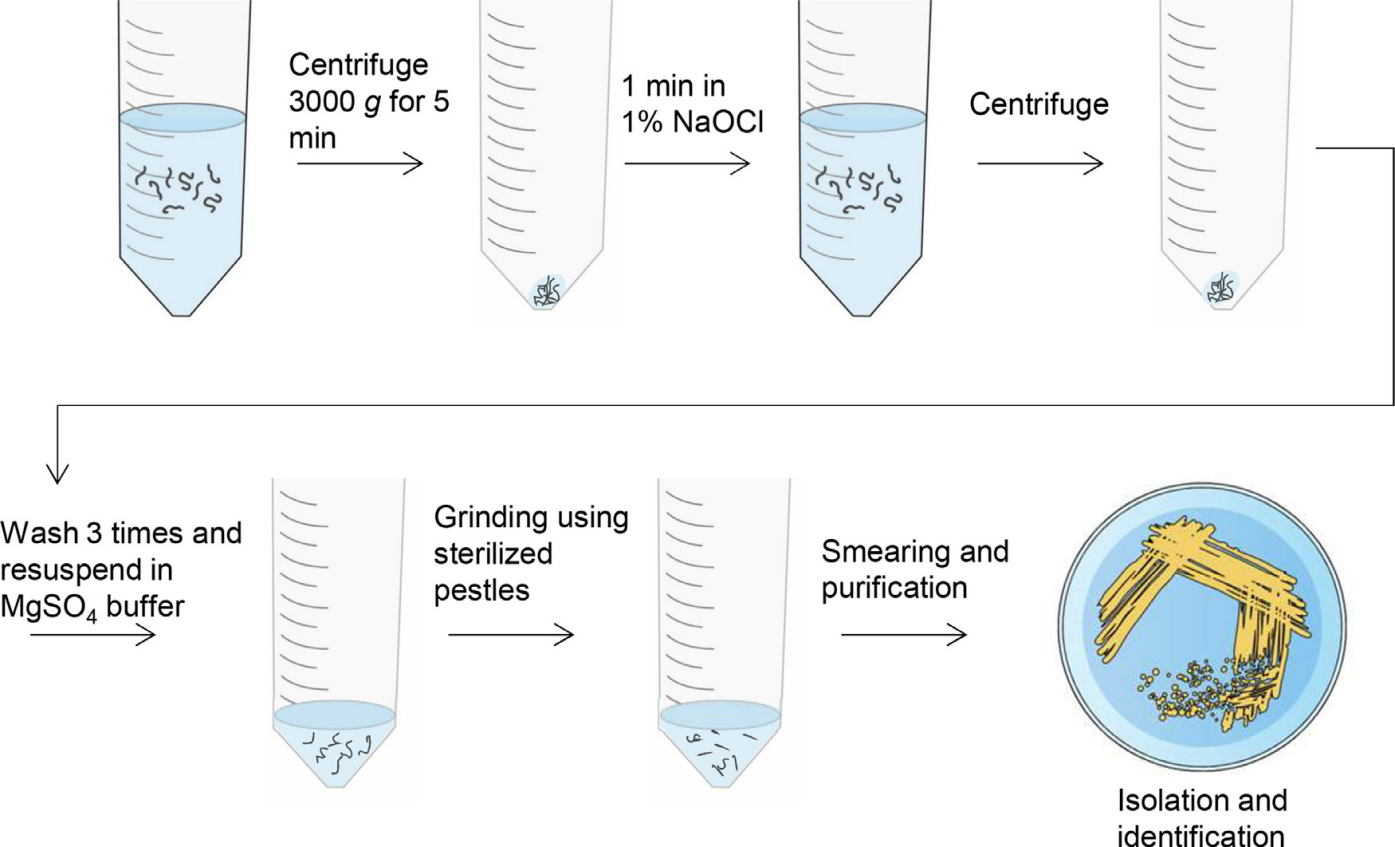
Schematic representation of endo-symbiotic bacterial isolation from nematodes that were isolated using the novel syringe method.

An illustration of the procedure on the surface sterilization and isolation of nematode endo-symbiotic bacteria are shown in Fig. 2.

### Safety precautions

• Nematodes, microbes, and chemicals must be carefully handled within a biosafety cabinet to prevent contamination. Properly dispose of biohazardous waste, including nematodes and microbial cultures.

### Method validation

**Fig. 3:**
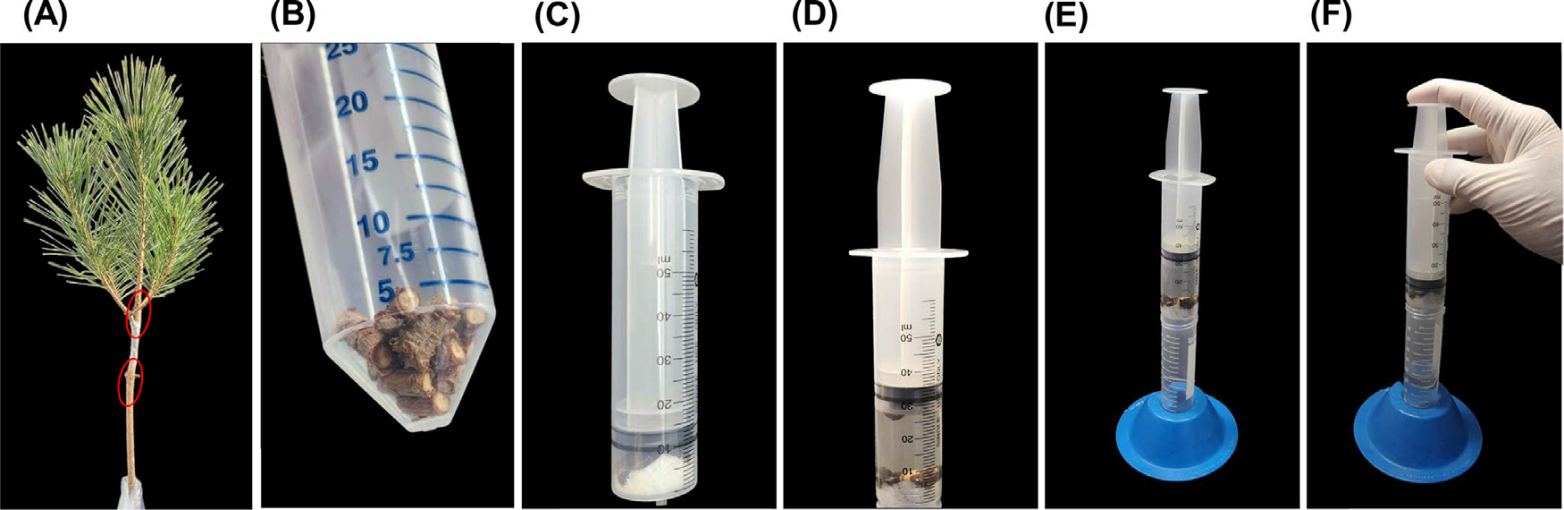
Nematode isolation from infected *Pinus densiflora* seedlings. (A) Photograph of inoculated seedlings. The red circle highlights the region from which samples were cut for nematode isolation. (B) After surface sterilizing the stem cuttings, they were further sectioned into smaller pieces of approximately 0.5 cm. (C) These pieces of stem cutting were placed into the barrel of a 50 mL sterile syringe, which had been pre-filled with multiple layers of autoclaved cheesecloth. (D) 25 mL of sterile 10 mM MgSO_4_ buffer was then drawn up using the syringe plunger. (E) The syringe was subsequently mounted on top of a Falcon tube and left undisturbed for 8 h at room temperature. This allows the nematodes to be released into the suspension. (F) The plunger is gently pressed to push the solution, along with the nematodes, into the Falcon tube. This solution is then ready for subsequent steps.

The protocol detailed herein was employed for the nematode isolation from infected seedling stem tissues (Fig. 3). The obtained nematodes were used for isolation and subsequent identification of bacterial strains that exist as endosymbionts within nematodes. Our surface sterilization of nematodes was confirmed to be effective as plating of unground nematodes on NA plates resulted in no visible bacterial colonies. However, upon grinding surface-sterilized nematodes under aseptic conditions, we isolated several bacterial strains. These strains were identified through partial 16S rRNA sequence analysis and the identified genera (*Acinetobacter, Bacillus, Pandoraea, Pantoea, Pseudomonas, Pseudoxanthomonas*) are mostly consistent with previous studies on nematode-symbiotic bacteria [3,10,11].

The validation of our method, was based on specific criteria to assess its effectiveness. Initially, after the isolation process, the extracted nematodes were meticulously examined under a microscope to confirm their presence and evaluate their viability. Subsequently, the bacterial strains that were isolated from these nematodes underwent a 16S rRNA sequence analysis. The genera identified through this process were then validated from previous studies as nematode-endosymbiotic bacteria.

Furthermore, we successfully utilized this new protocol to directly isolate nematodes from *in vitro* cultures maintained on *B. cinerea* PDA agar plates, yielding a viable nematode suspension ready for subsequent applications, which confirms the versatility of the new method.

This improved method provides a more streamlined, simplified and controlled process for the isolation of nematodes. It ensures less possibility of contamination and can be particularly beneficial for studies focused on nematode-associated microbes and their potential influence on nematode-associated microbiomes, given that the isolated nematodes could be used for metagenomic DNA isolation and analysis with a reduced risk of contamination compared to traditional methods.

One primary advantage of our novel nematode isolation technique over the conventional Baermann funnel method is the mechanism used to extract nematodes from wood tissue. For over a century, the Baermann funnel method has relied on the nematode natural movement, enabling them to migrate from the infected tissue and settle at the base of the funnel due to gravity. However, this method faces inherent limitations when isolating nematodes from wood tissues, especially during early infection stages, when nematode concentration is low or when they are in inactive lifecycle phases [12]. Our technique employs a syringe to apply mechanical pressure on the infected tissue, efficiently forcing out the nematodes for more thorough extraction. This method reduces dependency on the natural movement of nematodes, ensuring a consistently higher yield. While the traditional approach might necessitate extended periods (∼48 h) for optimal nematode extraction, our syringe-driven process accelerates the isolation, yielding more nematodes in less time. Moreover, the aseptic conditions preserved in our technique, along with the precise pressure application, mitigate contamination risks, ensuring the recovery of nematodes in their prime viable condition.

The implications of this method for PWD management are profound, as the deeper understanding of the nematode populations can lead to more targeted interventions. More importantly, this method offers the potential to provide deeper insights into the complex relationships between nematodes and their associated microbiota. The enhanced purity and efficiency of nematode isolation mean that subsequent genomic and microbiological analyses can proceed with a higher level of confidence. This could lead to important findings about how nematode-associated microbiota contribute to the progression and severity of PWD, or potentially control the nematode populations.

While our method has demonstrated efficiency and precision, there are potential limitations. These include the expenses associated with obtaining sterilized equipment, the limit of the syringe volume, and the preparatory step of autoclaving and sterilizing the cheesecloth before initiating the process. Potential future improvements could involve refining the syringe mechanism to handle larger tissue samples or creating a more adaptable setup. Additionally, designing a comprehensive set featuring built-in sterilized filters, as a replacement for the cheesecloth, would enhance the efficiency of the process.

In conclusion, we propose this enhanced protocol as a more controlled and efficient method for the aseptic isolation of nematodes. It guarantees aseptic conditions, rendering it particularly beneficial for studies aiming to evaluate the impact of various treatments on nematode endosymbionts and associated microbiota. The successful isolation and in-depth study of these nematodes can provide insights into the pathology of PWD, leading to better management and mitigation strategies. The method was validated through its successful application to infected pine seedlings. From these seedlings, nematodes were isolated, followed by the isolation of nematode-associated bacteria. These bacteria were then molecularly identified and compared with findings from previous research. Moreover, the proposed technique achieves faster and more efficient isolation of nematodes from wood tissues, making it particularly beneficial for various applications, including wood inspections at port quarantine centers.

## CRediT authorship contribution statement

**Mohamed Mannaa:** Conceptualization, Methodology, Visualization, Writing – original draft. **Young-Su Seo:** Conceptualization, Investigation, Writing – review & editing.

## Declaration of Competing Interest

The authors declare that they have no known competing financial interests or personal relationships that could have appeared to influence the work reported in this paper.

## Data Availability

No data was used for the research described in the article.
